# Intranodal lymphangiography with lipiodol as a diagnostic and therapeutic approach for spontaneous cervical chyle leak

**DOI:** 10.1002/ccr3.8161

**Published:** 2023-12-19

**Authors:** Yuki Sasaki, Yusuke Sakuhara, Satoru Sasaki, Taku Maeda, Yuhei Yamamoto, Kosuke Ishikawa

**Affiliations:** ^1^ Department of Plastic and Reconstructive Surgery, Faculty of Medicine and Graduate School of Medicine Hokkaido University Sapporo Japan; ^2^ Center for Vascular Anomalies, Department of Plastic and Reconstructive Surgery Tonan Hospital Sapporo Japan; ^3^ Department of Diagnostic and Interventional Radiology Tonan Hospital Sapporo Japan

**Keywords:** dermatology, radiology & imaging, vascular surgery

## Abstract

We present a case of spontaneous cervical chyle leak that showed as left‐sided neck swelling. Spontaneous chyle leak is extremely rare. Lymphangiography with lipiodol is useful as a diagnostic and therapeutic approach for chyle leak.

## Graphical abstract

Intranodal lymphangiography with lipiodol showed lymphatic leakage from near the venous angle to the left side of the neck (white arrow).

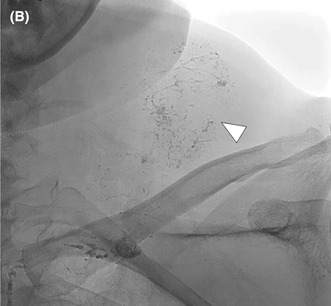

## 1 INTRODUCTION

Chyle leak is a condition in which the thoracic duct is damaged for some reason, causing the leakage of chyle from the lumen. The retention of the leaked chyle leads to chylothorax in the chest and chylous ascites in the abdomen.^1^, ^2^ Leakage of chyle into the subcutaneous layer can sometimes resemble a mass, making it difficult to differentiate from subcutaneous tumors or soft tissue tumors.^3^ Cervical chyle leak often occurs as a complication following procedures such as neck dissection or esophagectomy, due to the anatomical course of the thoracic duct.^4^, ^5^ On the contrary, spontaneous cervical chyle leak occurs without any clear cause such as surgery or trauma, and it is an extremely rare condition with very few reports.^6^, ^7^ Here, we report a case involving the successful diagnosis and treatment of cervical chyle leak by intranodal lymphangiography using lipiodol in a patient with a spontaneously occurring subcutaneous mass on the left side of the neck. The patient provided written informed consent for the report of her case details and imaging studies.

## 2 CASE REPORTS

**FIGURE 1:**
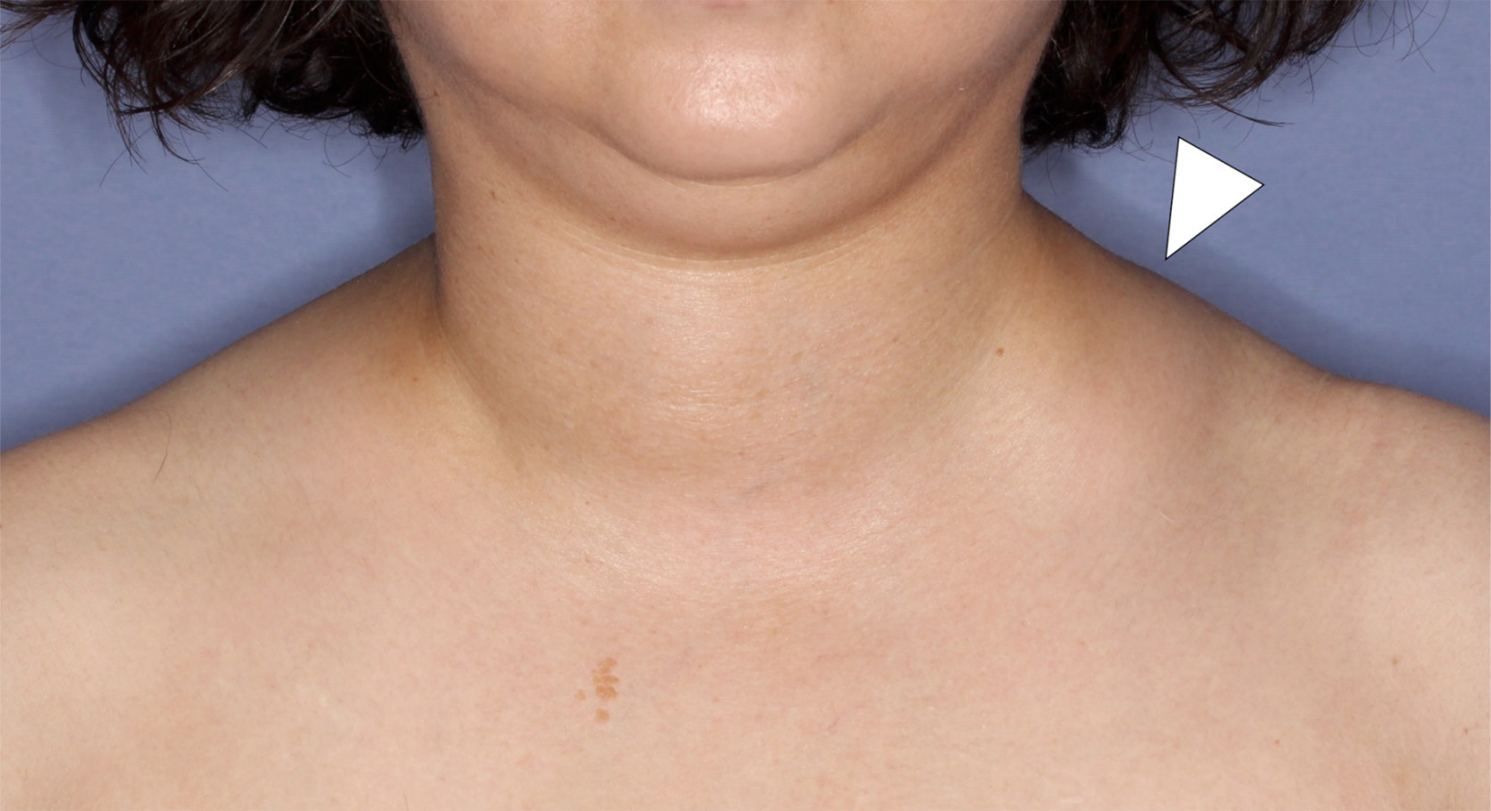
Clinical photograph obtained at the time of the patient's first visit to our department showing an 8 × 5‐cm elastic, soft, painless subcutaneous mass (white arrowhead) on the left side of the neck with no adhesions to the skin.

**FIGURE 2:**
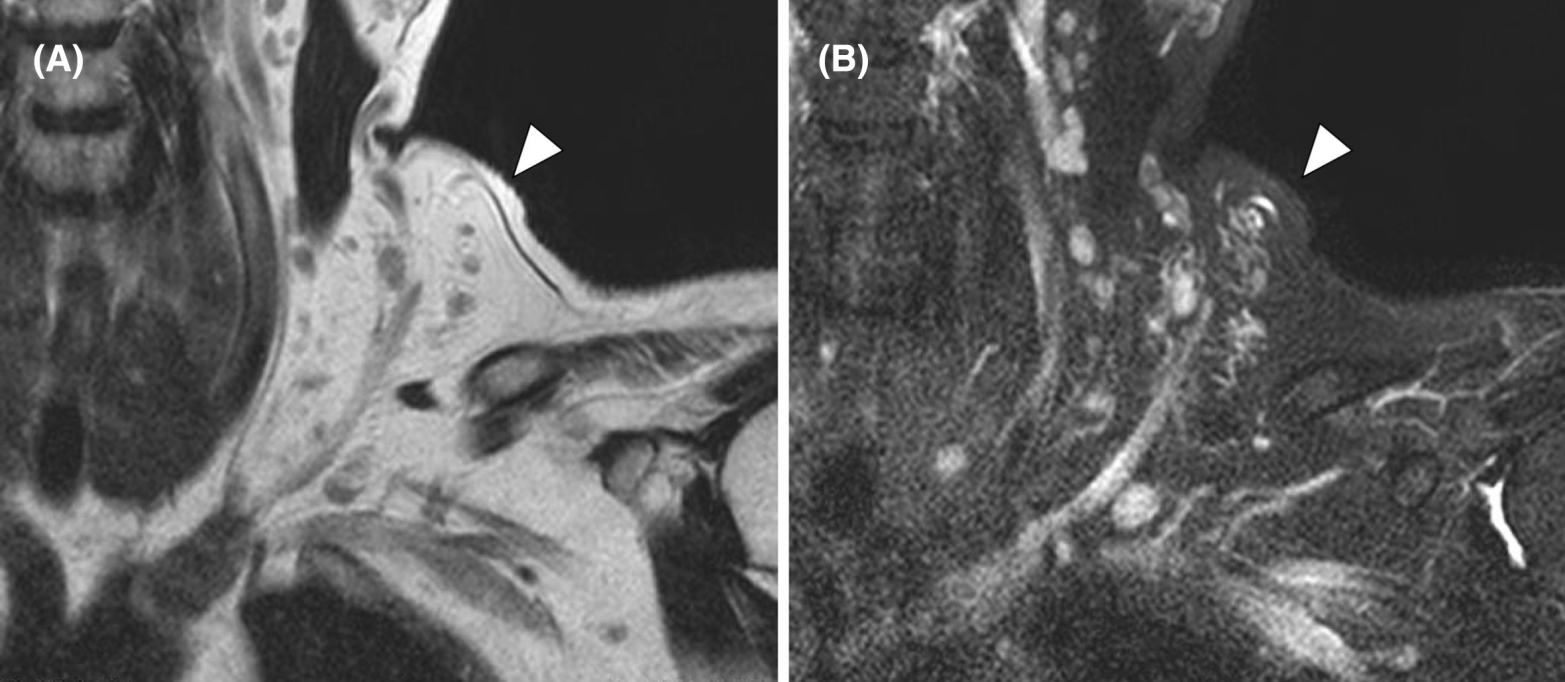
Coronal contrast‐enhanced magnetic resonance images. The lesion (arrowhead) had a borderline indistinct margin with (A) signal intensity equal to that of fat on T1‐weighted imaging and (B) high signal intensity on fat‐suppressed T2‐weighted imaging.

A 55‐year‐old woman with no medical history of note presented to another physician with left‐sided neck swelling that had appeared 5 years earlier in the absence of trauma or surgery (Figure 1). Contrast‐enhanced magnetic resonance imaging revealed an indistinct lesion with a signal equal to that of fat on T1‐weighted imaging (Figure 2A) and a high signal on fat‐suppressed T2‐weighted imaging (Figure 2B). The left external jugular vein coursed through the lesion, which extended deeply from the internal jugular vein to the posterior surface of the left sternocleidomastoid muscle. Based on these findings, a vascular malformation was suspected and the patient was referred to our department.

**FIGURE 3:**
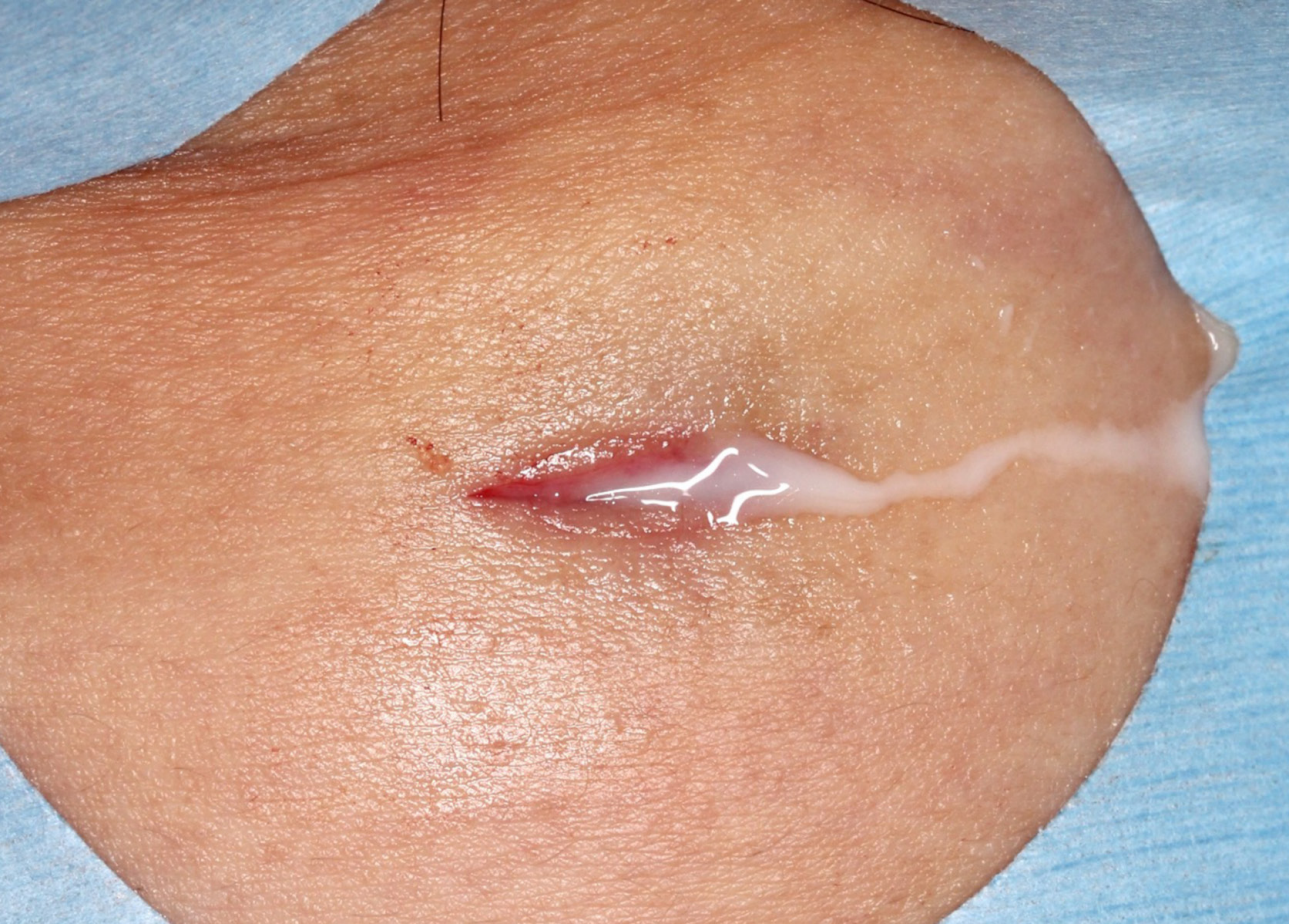
Clinical photograph obtained at the time of incisional biopsy showing milky white fluid flowing from the lesion.

At the initial visit, an 8 × 5‐cm, elastic, soft, painless subcutaneous mass was found on the left side of the neck. There were no adhesions to the skin. Ultrasonography revealed a subcutaneous mass with echoluminance comparable with that of fat. Considering the possibility of angiolipoma or venous malformation, an incisional biopsy was performed under local anesthesia. Milky white liquid then flowed out of the lesion (Figure 3).

**FIGURE 4:**
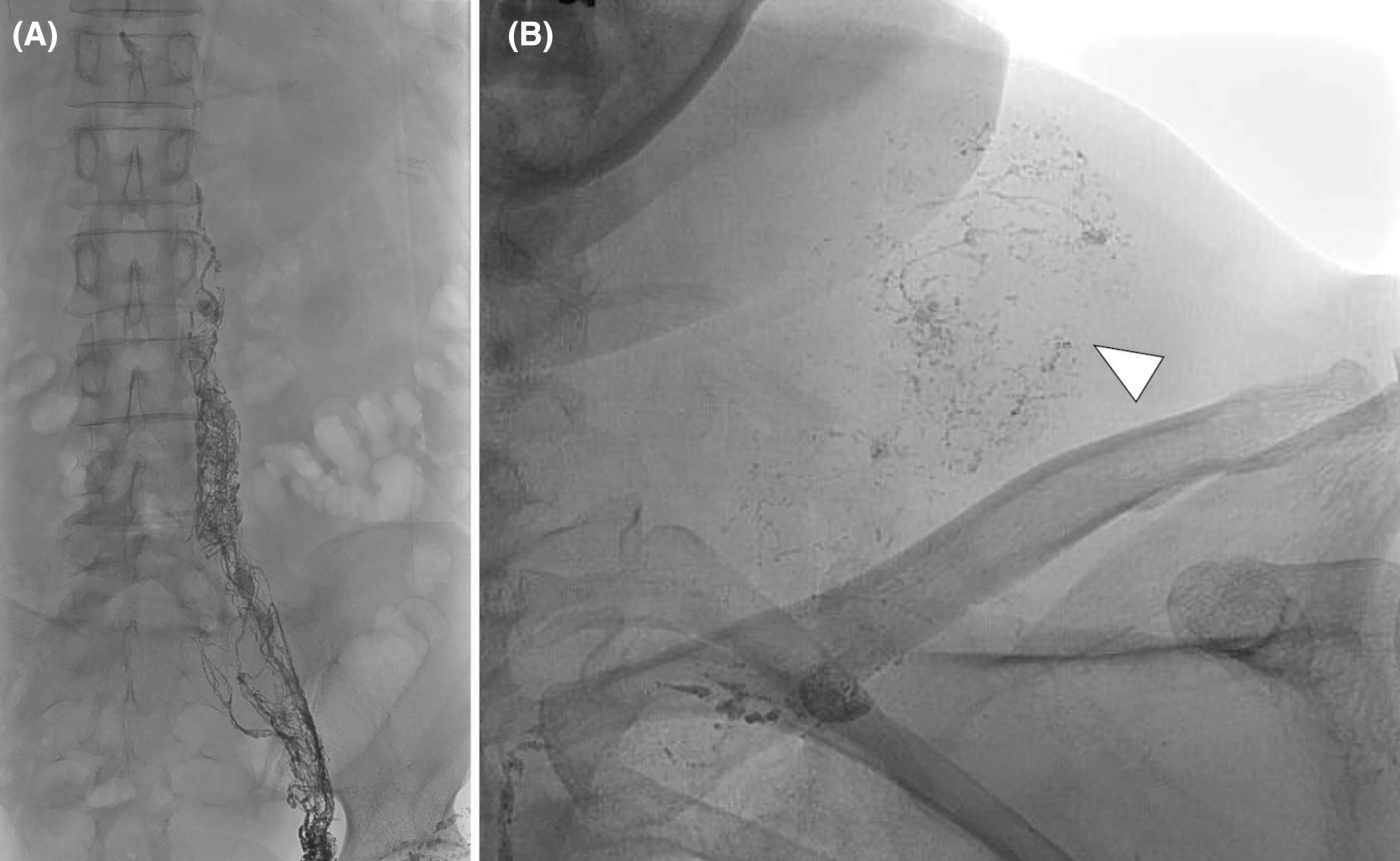
Intranodal lymphangiography using lipiodol images. (A) The inguinal lymph node was punctured to image the thoracic duct. (B) Lymphatic leakage from near the venous angle to the left side of the neck was observed (white arrowhead).

**FIGURE 5:**
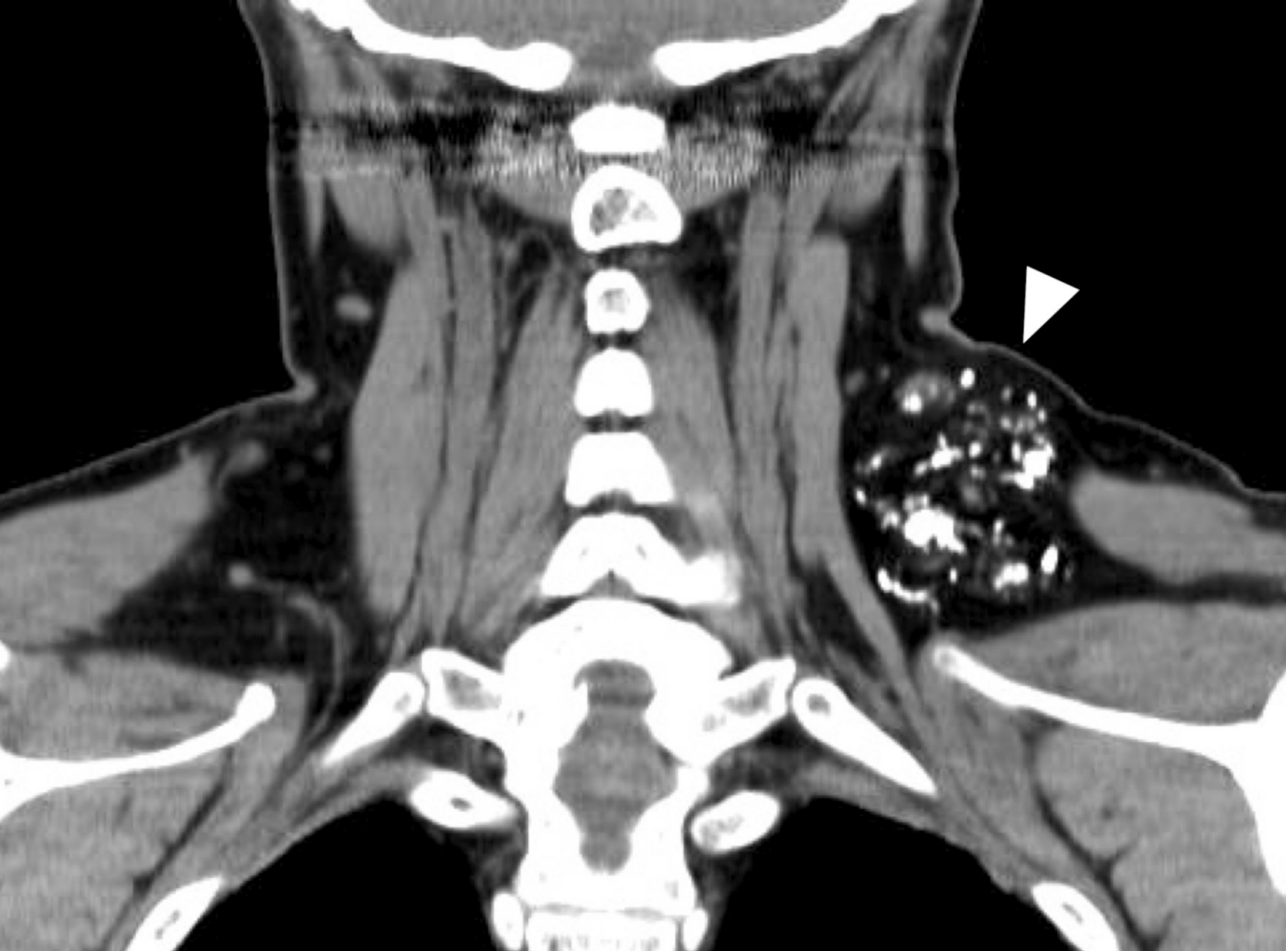
Computed tomography scan obtained after intranodal lymphangiography showing a hyperabsorptive zone within the lesion (white arrowhead) that was thought to represent lipiodol deposition.

Suspecting a chyle leak, the patient was referred to our diagnostic radiology department for intranodal lymphangiography. Lipiodol was used as the contrast medium. After puncturing a lymph node in the inguinal region, a catheter was advanced into the thoracic duct (Figure 4A). Abnormal lymphatic flow was detected from near the venous angle to the left side of the neck (Figure 4B). Considering that there was no history of surgery or trauma that could be the cause and no lymphatic leak such as chylothorax or chylous ascites elsewhere in the body, a diagnosis of spontaneous cervical chyle leak was made. Computed tomography was performed after intranodal lymphangiography and showed a hyperabsorptive zone within the lesion that was thought to represent deposition of lipiodol (Figure 5). The mass also appeared to be shrinking. Considering that the mass was not interfering with her day‐to‐day activities, no additional treatment was provided. However, she was followed up because of the possibility of infection.

## 3 DISCUSSION

Chyle is a milky white liquid composed of lymph fluid and triglycerides that is formed by the confluence of lymph fluid collected from the lower extremities and fat absorbed from the intestinal tract and flows into the thoracic duct.^8^ The thoracic duct collects lymphatic flow from the lower extremities, abdomen, and left upper body and returns it to the venous circulation at the venous angle.^8^ Cervical chyle leak usually occurs secondary to lymphatic obstruction after surgery, such as neck dissection, or trauma.^6^, ^7^, ^8^ To our knowledge, there have been only two cases of spontaneous cervical chyle leak reported to date.^6^, ^7^ In the previous two cases, chyle leak was suspected because of the presence of chyle leak through an incision. One case was treated by partial excision of the cystic chest duct,^6^ and the other was treated conservatively.^7^ This is the first report of spontaneous cervical chyle leak treated with intranodal lymphangiography with lipiodol alone.

If chyle leak is suspected, a systematic search using computed tomography or magnetic resonance imaging should be performed to check for complications, such as malignancy, chylothorax, or chylous ascites.^9^ Lymphoscintigraphy is useful for evaluating lymphatic function, including lymphatic leak and congestion, and may be used to diagnose chyle leak.^10^ Lymphangiography with injection of lipiodol is useful for understanding the structure of the lymphatic vessels, identifying the site of a leak, and treating it.^9^ There have been several reports on the usefulness of lymphangiography with lipiodol as a diagnostic and therapeutic approach for chyle leak.^11^, ^12^ Lipiodol is an oil‐based contrast agent used not only in lymphangiography but also in transcatheter arterial chemo‐embolization for hepatocellular carcinoma and in hysterosalpingograms in gynecology.^13^ Due to its iodine‐based nature, it should be administered cautiously to patients who may be allergic to iodine or who have thyroid disease.^13^ Lipiodol is thought to improve chyle leak by triggering a local inflammatory response that results in obstruction of the lymphatic vessels.^14^

## 4 CONCLUSIONS

We report a case of spontaneous cervical chyle leak. If a patient presents with subcutaneous mass on the left side of the neck without any specific trigger, spontaneous cervical chyle leak should be listed as a differential diagnosis. Lipiodol lymphangiography is a useful approach for diagnosis and treatment of chyle leak.

## AUTHOR CONTRIBUTIONS

**Yuki Sasaki:** Data curation; visualization; writing – original draft. **Yusuke Sakuhara:** Data curation; resources; visualization. **Satoru Sasaki:** Conceptualization; supervision. **Taku Maeda:** Investigation; visualization. **Yuhei Yamamoto:** Supervision; writing – review and editing. **Kosuke Ishikawa:** Conceptualization; project administration; writing – review and editing.

## FUNDING INFORMATION

No fund was available for this study.

## CONFLICT OF INTEREST STATEMENT

The authors declare no conflict of interest.

## ETHICAL APPROVAL STATEMENT

The patient has provided written informed consent for the publication of this case report.

## CONSENT

Written informed consent was obtained from the patient to publish this report in accordance with the journal's patient consent policy.

## Data Availability

The data that support the findings of this study are available from the corresponding author upon reasonable request.
